# Effects of Colostrum Source, Dose and Processing on *In Vitro* Rumen Fermentation in Weaned Lambs

**DOI:** 10.3390/ani16121761

**Published:** 2026-06-06

**Authors:** Jennifer Muñoz-Grein, Amina Hind Chekikene, Dimitra Graikini, Lourdes Sánchez, Manuel Fondevila, Alejandro Belanche

**Affiliations:** Departamento de Producción Animal y Ciencia de los Alimentos, Instituto Agroalimentario de Aragón (IA2), Universidad de Zaragoza, Miguel Servet 177, 50013 Zaragoza, Spain; jennifer.munoz@unizar.es (J.M.-G.); achekikene@unizar.es (A.H.C.); grekinid@gmail.com (D.G.); lousanchez@unizar.es (L.S.); mfonde@unizar.es (M.F.)

**Keywords:** bioactive compounds, immunoglobulins, rumen modulation, weaning

## Abstract

Young dairy ruminants often have limited rumen fermentative capacity, which can impair adaptation to solid diet during weaning. This study evaluated the effects of ruminant colostrum supplementation to enhance in vitro rumen fermentation at weaning. A batch culture system using rumen inoculum from artificially reared lambs was incubated with ovine, bovine, or reconstituted bovine colostrum at 4 and 20 mL/L. Sequential colostrum fractionation was used to identify the main functional components. Whole colostrum supplementation at high doses reduced ruminal pH (*p* < 0.001) and increased gas production (+18%), ammonia-N (+41%) and butyrate proportion (+10%) across colostrum sources, indicating enhanced microbial fermentation. Responses were dose-dependent and consistent across colostrum sources. Lipid removal had minimal effects, whereas depletion of high-molecular-weight molecules, mostly IgG, markedly reduced gas production and protein degradation. Removal of medium-size proteins such as lactoferrin produced minor changes. Residual activity in low-molecular-weight fractions was retained likely associated with the presence of fermentable substrates and bioactive compounds. These findings indicate that colostrum may exhibit both nutritional and modulatory effects on in vitro rumen fermentation, with IgG identified as a key component. These findings support the preservation of IgG in colostrum-derived products to enhance rumen fermentation, although further in vivo validation is required to evaluate the effects in weaned ruminants.

## Simple Summary

In intensive systems, young dairy ruminants are separated from their mothers at birth and fed artificial milk, which may impair rumen microbial development and hinder weaning transition. This study investigated whether colostrum supplementation could improve rumen microbial fermentation during the post-weaning period. Colostrum from sheep, cows, and a commercial bovine colostrum were tested at two doses using rumen fluid from artificially reared lambs. All colostrum sources enhanced microbial fermentation and increased butyrate production, a key compound for rumen development. Additional colostrum fractionation showed that removing fat had minor effects on rumen fermentation, while immunoglobulin G appeared to be the main component responsible for these positive effects. Overall, the results suggest that colostrum may enhance rumen fermentation both as a nutrient source and as a modulator of microbial activity, highlighting the potential use of immunoglobulin G-enriched colostrum products in young ruminant nutrition.

## 1. Introduction

In ruminant meat production systems, newborns suckle milk while progressively acquiring rumen microbiota and learned-feeding behavior from their dams, making weaning a natural transition from liquid to solid feed [1]. In contrast, in intensive dairy systems, newborn ruminants are commonly separated from their dams shortly after birth and artificially reared on milk replacer. The absence of contact with adult animals has been associated with delayed rumen microbial colonization, characterized by the absence of protozoa, reduced microbial diversity, and lower fermentative capacity [2]. Early weaning programs represent an additional challenge for artificially reared ruminants as their underdeveloped rumen microbial ecosystem must rapidly adapt to digest solid feeds with complex carbohydrates and fiber. These nutritional constraints, together with environmental and social stressors associated with weaning, can impair nutrient utilization, compromise growth performance, and increase susceptibility to post-weaning diarrhea [2,3].

Stimulating rumen fermentation during the post-weaning period has been proposed as a key strategy to optimize productivity in artificially reared ruminants [1]. Rumen fermentation produces volatile fatty acids (VFAs), which can supply up to 70% of the metabolizable energy requirements in ruminants [4]. Increased VFA production also contributes to a reduction in ruminal pH, potentially limiting the proliferation of opportunistic pathogens such as *Escherichia coli* and reducing the incidence of post-weaning diarrhea. In addition, butyrate serves as a primary energy source for rumen epithelial cells and plays a critical role in the development of rumen papillae, which may undergo transient atrophy during the weaning transition [4].

Ruminant colostrum and derivatives, including whole frozen colostrum, skimmed colostrum, colostrum powders, whey-derived ingredients, immunoglobulin-rich concentrates, and formulated replacers or supplements, are considered high-value commercial products and have gained increasing attention because of their functional properties. Colostrum products have a high concentration of bioactive compounds, including immunoglobulins (mainly IgG), antimicrobial proteins (e.g., lactoferrin, lactoperoxidase, and lysozyme) and peptides, growth factors (e.g., insulin-like growth factor-1, insulin, transforming growth factor-β, and cytokines), and oligosaccharides, which are essential in young ruminants [5,6]. In intensive livestock systems, colostrum-derived products are widely used as sources of IgG to enhance passive immune transfer to the newborn [7], but they may also be relevant during the weaning transition, as they can improve gut integrity, reduce intestinal permeability, and enhance growth performance in both piglets [8] and calves [9]. Although some colostrum components, such as IgG, can partially resist gastrointestinal digestion, the potential use of colostrum-derived products as feed additives specifically aimed at modulating rumen fermentation during weaning remains poorly understood.

Colostrum industrial processing into functional ingredients has considerably advanced in recent years through technologies such as spray-drying, membrane filtration, and protein fractionation, enabling the development of nutraceuticals and feed additives with targeted biological functions [10]. However, different colostrum bioactive compounds vary in their sensitivity to inactivation during processing, leading to reduced biological activity and greater variability in efficacy among commercial products. Therefore, identifying the specific colostrum components that positively influence rumen fermentation during post-weaning is essential to guide the development of such fractionation and processing technologies.

The aim of this study was to evaluate the potential of ruminant colostrum to support rumen fermentation during the post-weaning transition in artificially reared lambs. It was hypothesized that colostrum supplementation could exert a dual beneficial effect by providing both fermentable nutrients and bioactive molecules. To test this hypothesis, an in vitro incubation using rumen fluid from artificially reared lambs was employed to assess the effects of increasing doses of three colostrum sources on rumen microbial fermentation. Additionally, a fractionation approach was applied to identify the specific colostrum components associated with the observed responses.

## 2. Materials and Methods

### 2.1. Colostrum Isolation and Fractionation

Ovine colostrum (OC) was obtained from the first milking of 30 Assaf sheep (Santa Eulalia, Spain), pooled and immediately frozen at −20 °C. Bovine colostrum (BC) and reconstituted bovine colostrum (RBC) were supplied by Phytobiotics Futterzusatzstoffe GmbH (Eltville am Rhein, Germany). Bovine colostrum was obtained from the first milking from German dairy farms, and quality control was performed by the manufacturer to verify the IgG content and the absence of antibiotic residues before it was pooled and frozen at −20 °C. A part of BC from the same batch was defrosted at 5 °C, homogenized, pasteurized at 60 °C for 60 min, vacuum-dried and stored at 4 °C in airtight bags as a commercial product (Immune Milk^®^, Phytobiotics Futterzusatzstoffe GmbH, Eltville am Rhein, Germany). The RBC used in this study was obtained by mixing 1 g of Immune Milk^®^ and 4 mL of water at 38 °C, following the manufacturer’s instructions, in order to obtain RBC with similar DM to that observed in BC.

**Figure 1:**
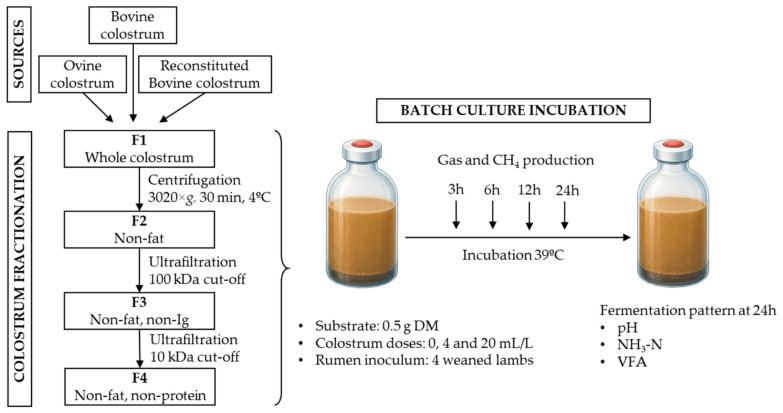
Illustration of the colostrum sources, fractionation procedure and in vitro incubation.

All three colostrum sources (OC, BC and RBC) were sequentially fractionated into four fractions (Figure 1): Fraction 1 (F1) was the whole colostrum; Fraction 2 (F2) was the defatted or non-fat fraction obtained after centrifugation of F1 at 5000× *g* for 30 min at 4 °C to remove the upper fat layer; Fraction 3 (F3) was the non-fat, non-IgG fraction which was obtained as the permeate after ultrafiltration of F2 at the same speed using a 100 kDa cut-off ultrafiltration column (amicon Ultra-15, UFC910008, Merck, Germany). Finally, fraction 4 (F4) was the non-fat, non-protein fraction obtained as the permeate after ultrafiltration of F3 at the same speed using a 10 kDa cut-off column (amicon Ultra-15, UFC901024, Merck, Germany) to remove medium- and small-size proteins but retaining oligosaccharides and bioactive peptides. All fractions were frozen until the incubation.

### 2.2. In Vitro Batch Fermentation

A batch culture incubation was performed to evaluate the effects of different types and doses of colostrum on rumen fermentation, mimicking a post-weaning scenario. Rumen inocula were obtained from four 8-week-old Assaf male lambs (13.2 ± 2.16 kg body weight) raised in the same commercial farm that provided the OC (Santa Eulalia, Spain). Lambs were separated from their dams immediately after birth and reared artificially, with *ad libitum* access to milk replacer, concentrate feed, and barley straw. Lambs were slaughtered one week after weaning (8 weeks of age) at a commercial abattoir (Cella, Teruel, Spain). Rumen contents from each lamb were filtered through two layers of muslin and transferred to the laboratory in thermos flasks. Before incubation, rumen contents from each lamb were diluted at a 1:4 ratio with an anaerobic incubation buffer [11] in which the bicarbonate concentration was adjusted to achieve a target incubation pH of 6.4 [12]. Inoculum pH was measured and samples were collected for ammonia-N (NH_3_-N), VFA and protozoal counting as described below.

A factorial 3 × 2 dose–response incubation was conducted for each type of colostrum (OC, BC and RBC) considering four fractions (F1, F2, F3 and F4) and two doses (4 and 20 mL/L), as well as the control (CTL) without colostrum supplementation. A batch culture incubation was performed into 120 mL Wheaton bottles containing 50 mL of incubation solution and 500 mg DM of substrate. This experimental substrate was formulated as a mixed diet to meet the nutrient requirements of lambs at weaning and included (in FM) 27.6% barley, 18.4% wheat, 18.4% maize, 9.2% soybean meal, 9.2% wheat bran, 4.6% sunflower seeds, 4.6% distiller’s dried grains with solubles and 8% barley straw. Substrate composition (g/kg DM) was: organic matter (OM, 959), crude protein (CP, 159), ether extract (EE, 29.2), neutral detergent fiber (NDF, 261), acid detergent fiber (ADF, 114), and acid detergent lignin (ADL, 15.4). The incubation was conducted with rumen inocula from 4 independent lambs for each treatment without technical replication, and additional bottles including substrate-blanks (bottles supplemented with colostrum fractions at 20 mL/L but without substrate) and blanks (bottles without colostrum and substrate) were included.

The incubation process was carried out under anaerobic conditions at 39 °C for 24 h. Gas pressure in the headspace was measured for 3, 6, 12 and 24 h using a manometer (DH 2124.02 Delta Ohm, Caselle di Selvazzano, Italy), and converted to gas production (GP) volume using the ideal gas law. In addition, gas samples (4.5 mL) were collected after reading at 3 and 24 h to determine the methane (CH_4_) concentration using gas chromatography (Agilent 6890 Series GC System, Santa Clara, CA, USA) equipped with a flame ionization detector and a capillary column (HP-1, 30 m × 530 μm id × 1.5 µm film thickness) and using He as gas carrier. After 24 h of incubation, bottles were opened, and pH was recorded using a CRISON micro-pH meter 2001 (Barcelona, Spain) and two 1 mL samples were collected. One was mixed with 0.25 mL of H_3_PO_4_ buffer (0.5 mol/L) containing 4-methyl valeric acid as internal standard (2 g/L) for volatile fatty acid (VFA) analysis using the same gas chromatography apparatus and gas carrier as for the CH_4_, with a capillary column (HP-FFAP Polyethylene glycol TPA, 30 m × 530 μm id × 1 µm film thickness). The second sample was mixed with 0.5 mL of HCl (3 mol/L) for NH_3_-N determination using a colorimetry method.

### 2.3. Laboratory Analyses

The chemical composition of the substrate was determined following the AOAC procedure [13] for dry matter (DM, method 934.01), organic matter (OM, method 942.05), crude protein (CP, method 976.05), acid detergent fiber (ADF), acid detergent lignin (ADL, method 973.18), and ether extract (EE, method 2003.05). Fiber concentration was determined as described by Mertens [14] using an Ankom 200 Fiber Analyzer (Ankom Technology, New York, NY, USA), and results were expressed without residual ashes. The total starch concentration was measured enzymatically (Total Starch Assay Kit K-TSTA 07/11, Megazyme, Bray, Ireland).

The colostrum composition was determined in three samples per fraction using a milk analyzer (Lactoscan SP+, Milkotronic Ltd., Nova Zagora, Bulgaria). Concentration of IgG in colostrum was measured using different approaches: a digital Brix refractometer (Deltatrak, Pleasanton, CA, USA) with temperature correction was used to estimate the IgG concentration using the following equation IgG = −61.896 + 5.666 × BRIX [15]. The sheep IgG concentrations in the OC fractions and the bovine IgG in the BC and RBC fractions were quantified using ELISA kits (CSB-E14400Sh and CSB-E1205B, respectively. Cusabio^®^, Houston, TX, USA). Concentration of lactoferrin, milk fat globule epidermal growth factor 8 (MFGE8), insulin-like growth factor I (IGF-I) and bovine epidermal growth factor (EGF) were determined using ELISA kits (MBS704467, MBS103771, MBS7606345 and MBS005514, respectively) according to the manufacturer’s protocols (MyBioSource Inc., San Diego, CA, USA) and the absorbance of all analytes was measured at 450 nm using a microplate spectrophotometer. Protein concentrations in colostrum fractions were determined using the bicinchoninic acid assay (ThermoFisher Scientific), with bovine serum albumin as standard and phosphate-buffered saline serving as diluent. Absorbance was measured at 560 nm using a microplate reader. All samples were assayed in quadruplicate.

In order to illustrate the effects of the fractionation on the protein profile, colostrum proteins were separated by their molecular weight using a sodium dodecyl sulphate–polyacrylamide gel electrophoresis (SDS-PAGE) according to Laemmli, [16]. Samples were diluted 1:1 with 0.12 M Tris-HCl buffer (pH 6.8) containing 20% (*v*/*v*) glycerol, 4% (*w*/*v*) SDS, and 0.02% (*w*/*v*) bromophenol blue. For analyses under reducing conditions, β-mercaptoethanol was added to a final concentration of 10% (*v*/*v*), and all samples were subsequently heated at 100 °C for 5 min to ensure complete protein denaturation. An electrophoretic separation was carried out on 4–20% precast polyacrylamide gels (Mini-PROTEAN Tetra Cell system, Bio-Rad Laboratories, Hercules, CA, USA) using a Pre-stained Protein Ladder PageRuler™ (ThermoFisher Scientific, Waltham, MA, USA) spanning from 10 to 180 kDa as a molecular weight reference. Gels were run at a constant current of 180 mA for 35 min. Following electrophoresis, proteins were visualized by staining with Coomassie Brilliant Blue R-250 (Serva Blue R, Serva Feinbiochemica GmbH & Co., Heidelberg, Germany).

### 2.4. Calculations and Statistical Analyses

Data were analyzed using SPSS software (IBM Corp. Version 21.0, New York, NY, USA). Each lamb inoculum was used as one independent biological replicate for every treatment. Data from blank and substrate-blank bottles were not included in the final calculations as they showed similarly low values. Rumen fermentation end-products were expressed both as final concentrations and as production per unit of substrate. The latter was calculated using the initial and final concentrations (after 24 h), the incubation volume in the inoculum, and the amount of substrate, which included both the diet and the colostrum, as follows: Production = (final concentration—initial concentration) × incubation volume/(diet + colostrum). A Shapiro–Wilk test was conducted prior to the analysis to assess the normality of the variables, and no significant deviations from normality were observed (*p* > 0.05). An initial ANOVA was performed to assess the effects of the three whole colostrum sources against the CTL for each incubation dose as follows:

*Y_ij_* = *µ* + *C_i_* + *A_j_* + *e_ij_*

where *Y_ij_* is the dependent, continuous variable, *µ* is the overall population mean, *C_i_* is the fix effect of the whole colostrum type (*i* = CTL vs. OC vs. BC vs. RBC), *A_j_* is the random effect of the lamb used as rumen inoculum (*j* = 1 to 4), and *e_ij_* is the residual error.

A 4 × 2 factorial ANOVA was further conducted for each colostrum type to describe the effects of the fractionation procedure and colostrum doses as follows:

*Y_klj_* = *µ* + *F_k_* + *D_l_* + (*F* × *D*)*_kl_* + *A_j_* + *e_klj_*

where *F_k_* is the fix effect of the of colostrum fractionation (*k* = F1 vs. F2 vs. F3 vs. F4), *D_l_* is the fix effect of the dose (*l* = 0.4% vs. 2% in volume), (*F* × *D*)*_kl_* represents the interaction term, and *Y_klj_*_,_ *µ*, *Aj* and *e_klj_* are the same terms as described before. When significant effects were detected, means were compared using Fisher’s protected LSD test in order to maximize the sensitivity. Moreover, pairwise comparisons were conducted between each treatment mean and the CTL considering the random effect of the lamb. Significant effects were declared at *p* < 0.05, and trends were considered when 0.05 ≤ *p* < 0.10.

## 3. Results

### 3.1. Colostrum Composition and Fractionation

The chemical composition of whole OC, BC, and RBC remained within physiological ranges (Table 1). Whole OC (F1) exhibited higher fat and IgG concentrations compared with BC and RBC. These differences in IgG concentration were consistently confirmed by both ELISA and Brix refractometry. However, IgG values estimated by Brix refractometry were systematically higher than those obtained by ELISA, corresponding to overestimations of 1.56-, 2.43-, and 2.60-fold for OC, BC, and RBC, respectively. Total protein, MFGE8, and EGF concentrations were comparable among the three whole colostrum sources. In contrast, IGF-I concentration was greater in OC than in BC or RBC, whereas lactose concentration showed the opposite pattern, being lower in OC than in BC. Overall, only minor compositional differences were observed between BC and RBC, consistent with their common origin from the same colostrum source.

**Figure 2:**
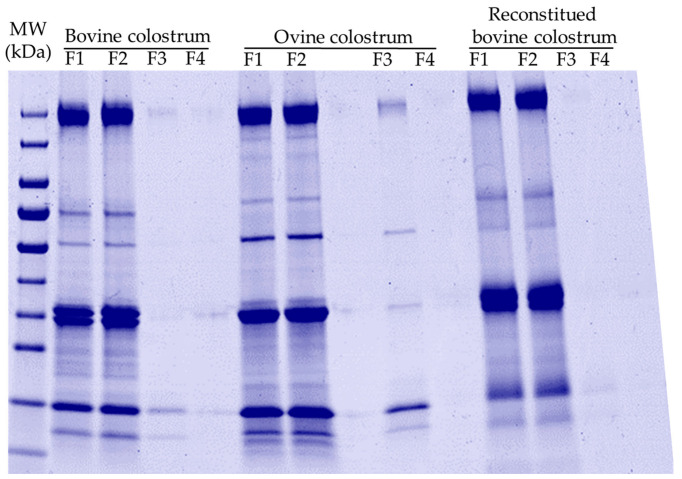
Effect of colostrum fractionation on the protein profile based on a sodium dodecyl sulphate–polyacrylamide gel electrophoresis (SDS-PAGE). F1, whole colostrum; F2, non-fat colostrum; F3, non-fat, non-IgG colostrum; F4, non-fat, non-protein colostrum.

Regarding colostrum fractionation, the centrifugation-based defatting step (F1 to F2) was highly effective, reducing fat content to non-detectable levels without affecting the concentration of the remaining components (Table 1 and Figure 2). Subsequent filtration of F2 through a 100 kDa cut-off membrane resulted in a marked reduction in total protein concentration, particularly affecting IgG levels across all colostrum types. Further filtration of F3 through a 10 kDa cut-off membrane substantially reduced the concentration of medium-sized molecules, including lactoferrin and MFGE8, while preserving smaller peptides such as EGF.

The compositional differences among fractions were further visualized by SDS-PAGE protein profiling (Figure 2). Electrophoretic analysis revealed similar banding patterns among F1 samples from OC, BC, and RBC, with prominent bands detected at approximately 180, 70, 55, 35, 15, and 12 kDa. Fraction F2 exhibited a protein profile comparable to that of F1 across all colostrum sources, indicating that defatting had minimal impact on protein composition. In contrast, F3 showed a marked reduction in band intensity, characterized by the absence of proteins above 100 kDa while retaining bands at approximately 55, 35, 15, and 12 kDa. The 180 kDa band observed in OC-F3 was likely attributable to a methodological artifact caused by IgG contamination from an adjacent well, as this band was not observed in BC-F3 or RBC-F3. Finally, F4 showed no prominent bands, confirming the effectiveness of the 10 kDa filtration step in removing the majority of protein components.

### 3.2. Effects of Different Sources of Whole Colostrum on In Vitro Rumen Fermentation

Microscopic examination confirmed the absence of rumen protozoa in the inocula obtained from the four artificially reared lambs used as donors. Initial ruminal pH (6.67 ± 0.02) and concentrations of NH_3_-N (85.1 ± 4.87 mg/dL) and total VFAs (159 ± 36.6 mmol/L) in the inocula were comparable to those observed at the end of the incubation (6.21 ± 0.06, 68.2 ± 12.2 mg/dL and 127 ± 25.3 mmol/L, Table 2).

The effects of whole colostrum from different sources on rumen fermentation were evaluated by incubating rumen fluid from weaned lambs for 24 h in batch cultures (Table 2). All whole colostrum sources similarly stimulated microbial fermentation activity. Supplementation with low doses of BC or RBC increased GP compared with the CTL, whereas the highest dose produced a more pronounced increase (*p* < 0.001). Moreover, GP was consistently greater with OC than with BC or RBC, regardless of the inclusion level (*p* = 0.001). Methane production also increased relative to the CTL following supplementation with either low (*p* = 0.006) or high doses (*p* < 0.001) of whole colostrum, with comparable responses across colostrum sources.

Regarding fermentation characteristics, ruminal pH was unaffected at the low inclusion level but decreased significantly at the high dose compared with the CTL (*p* = 0.001). Total VFA concentration and the molar proportions of acetate and propionate were not modified by colostrum supplementation. In contrast, the molar proportion of butyrate increased following supplementation with high doses of BC and RBC (*p* = 0.036). Low doses of whole colostrum increased NH_3_-N concentration (*p* < 0.001) and the molar proportions of isobutyrate (*p* = 0.044) and isovalerate (*p* = 0.086) relative to the CTL. These effects were further enhanced at the high dose (*p* < 0.001, *p* = 0.001, and *p* = 0.002, respectively), suggesting increased proteolytic activity. Notably, OC increased NH_3_-N concentration to a greater extent than BC or RBC.

When fermentation end-products were corrected for their initial concentrations in the inoculum and for substrate availability, some treatment effects were attenuated. Low doses of whole colostrum did not affect GP, total VFA concentration, or the molar proportions of major VFAs, but increased NH_3_-N (*p* = 0.049) and isovalerate production (*p* = 0.075) per unit of substrate, with similar responses across colostrum sources. In contrast, high doses of RBC reduced GP (*p* < 0.001) and acetate production (*p* = 0.045), but increased NH_3_-N (*p* = 0.016), isobutyrate (*p* = 0.053), valerate (*p* = 0.008), and isovalerate production per unit of substrate (*p* = 0.069).

### 3.3. Effects of Colostrum Fractionation and Dosage on in Vitro Rumen Fermentation

**Figure 3:**
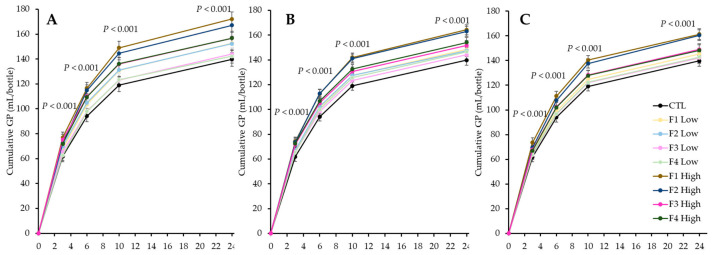
Effects of incubation with low (4 mL/L) or high (20 mL/L) doses of different fractions of ovine (**A**), bovine (**B**) or reconstituted bovine colostrum (**C**) on in vitro gas production. F1, whole colostrum; F2, non-fat colostrum; F3, non-fat, non-IgG colostrum; F4, non-fat, non-protein colostrum.

Cumulative in vitro GP increased with increasing colostrum dose and progressively decreased following colostrum fractionation, with these effects being consistent across colostrum sources (Figure 3). The greatest GP values were observed with F1 and F2 at the high dose (*p* < 0.001), followed by high-dose F3 and F4 and low-dose F1 and F2 treatments. In contrast, low doses of F3 and F4 resulted in GP values comparable to CTL.

Marked differences in rumen fermentation were detected among colostrum fractions and inclusion levels, whereas differences among colostrum sources were generally negligible (Table 3). Overall, supplementation with high doses of colostrum stimulated rumen fermentation, as reflected by increased GP, CH_4_ production, NH_3_-N concentration, and total VFA concentration, together with a reduction in ruminal pH compared with low-dose supplementation. Higher inclusion levels also shifted the fermentation profile toward greater molar proportions of isobutyrate, valerate, and isovalerate, and lower propionate proportions (Table 4). However, most of these responses were modulated by the interaction between fraction and dose, and to a lesser extent by colostrum source. For example, increasing the dose of RBC increased acetate proportion (*p* = 0.004) and decreased butyrate proportion (*p* = 0.022), whereas this effect was not observed for OC or BC.

Regarding the fractionation procedure, colostrum defatting (F1 to F2) had minor effects on rumen fermentation (Table 4). Similar stimulatory responses relative to the CTL were observed with F2 and F1, as described in Table 2. The most evident effect of defatting was an increase in ruminal pH for OC (*p* = 0.001) and RBC (*p* = 0.049) at the high dose. Consequently, F2 supplementation promoted lower ruminal pH and greater GP, CH_4_ production, NH_3_-N concentration, and total VFA concentration (for BC and RBC only), together with increased molar proportions of butyrate, isobutyrate, valerate, and isovalerate relative to the CTL. These effects were more pronounced at high than at low doses.

Filtration through the 100 kDa membrane (F2 to F3) induced marked changes in rumen fermentation. These effects were generally consistent across colostrum sources and more evident at the high inclusion level, as indicated by significant Fraction × Dose interactions. This filtration step reduced GP, CH_4_ production, pH, NH_3_-N concentration, and the molar proportions of isobutyrate, valerate, and isovalerate. In addition, the 100 kDa filtration increased the molar proportion of propionate in OC (*p* = 0.002) and RBC (*p* = 0.052). As a consequence, several of the stimulatory effects observed with F2 disappeared after this filtration step. Nevertheless, F3 supplementation still maintained some effects relative to the CTL, including lower pH and greater GP, CH_4_ production, and butyrate proportion, particularly at the high inclusion level (Fraction × Dose interaction). Furthermore, low doses of F3 from OC and RBC increased isobutyrate and isovalerate proportions compared with the CTL.

Further filtration through the 10 kDa membrane (F3 to F4) produced only minor additional changes in rumen fermentation. The most notable effects were reductions in the molar proportions of isobutyrate (*p* < 0.001) and isovalerate (*p* = 0.001) for high doses of OC-F4. Overall, F4 supplementation retained some modest positive effects on rumen fermentation, including lower pH and higher butyrate proportion relative to the CTL, as well as increased GP and CH_4_ production at the high inclusion level.

When fermentation end-products were expressed per unit of substrate, several effects of colostrum supplementation disappeared or were attenuated, with similar trends across colostrum sources (Table 5).

After correction, low colostrum doses resulted in greater GP and lower NH_3_-N production per unit of substrate than high doses, with these responses modulated by the Fraction × Dose interaction. Colostrum defatting increased GP and NH_3_-N production per unit of substrate, although these effects were only evident at the high dose. Due to the elevated protein concentration in F2, supplementation with this fraction increased NH_3_-N, isobutyrate, valerate, and isovalerate production, while reducing GP per unit of substrate relative to the CTL, particularly at high inclusion levels.

Removal of large proteins through 100 kDa filtration decreased NH_3_-N, isobutyrate, valerate, and isovalerate production per unit of substrate, with similar effects across colostrum sources. This filtration step also increased GP and CH_4_ production per unit of substrate, although only at the high dose. In contrast, further filtration through the 10 kDa membrane had negligible effects on fermentation outputs per unit of substrate. Consequently, supplementation with F3 or F4 generally resulted in values similar to those of the CTL. The only consistent differences were increased CH_4_ production and decreased NH_3_-N production relative to the CTL when F3 or F4 were supplemented at the high dose.

## 4. Discussion

Our experimental design aimed to mimic post-weaning conditions in artificially reared ruminants. Accordingly, rumen inoculum was collected from artificially reared lambs fed high-concentrate diets. These inocula were devoid of rumen protozoa, a situation commonly observed in non-mothered lambs [2]. This aspect is particularly relevant and can modulate the effectiveness of nutritional interventions, as protozoa play a key role in fiber degradation and contribute to butyrate and CH_4_ production [17]. Moreover, artificially reared lambs typically exhibit lower rumen bacterial diversity and reduced fermentative capacity compared to naturally reared counterparts, making the weaning transition more challenging [2]. The incubation substrate consisted predominantly of concentrate (92%), resulting in relatively high total VFA concentrations (126 ± 20.9 mmol/L) and moderately low pH values (6.21 ± 0.06). These conditions are comparable to those reported in weaned lambs (VFAs: 96–160 mmol/L; pH: 6.12–6.45) [18,19], indicating that the in vitro system reproduced physiological rumen fermentation conditions. Despite these similarities, caution is required when extrapolating in vitro results to in vivo animal physiology.

### 4.1. Effect of Whole Colostrum on In Vitro Rumen Fermentation

The composition of the three whole colostrum sources (F1) fell within physiological ranges [20,21]. Despite compositional differences among colostrum sources, particularly the lower fat and IgG concentrations observed in BC compared with OC, all sources elicited comparable effects on rumen fermentation in vitro. This suggests that the overall fermentative response was primarily driven by the general nutrient contribution of colostrum rather than by species-specific differences between sources. From a practical perspective, this supports the potential interchangeability of ovine and bovine colostrum, with RBC representing a convenient alternative due to its ease of storage and handling [7].

The increases in GP (up to +9% and +22% at low and high doses, respectively) and CH_4_ production (up to +21% and +24%, respectively), together with the reduction in ruminal pH, indicate an overall stimulation of microbial activity following colostrum supplementation. Importantly, these responses were proportional to the level of inclusion, suggesting that whole colostrum mainly acted as an additional fermentable substrate. The absence of differences in total VFA production per unit of substrate among treatments further supports this interpretation, indicating that fermentation efficiency was not substantially altered but rather that total fermentation was mostly increased due to greater substrate availability [22].

Nevertheless, the observed shift in fermentation pattern, particularly the increase in butyrate proportion (OC: +7%; BC: +13%; RBC: +16%) when different colostrum sources were supplemented at high doses suggests a more complex response that cannot be explained solely by nutrient supply. Lactose and oligosaccharides present in colostrum are known to serve as fermentable substrates for rumen microbes resulting in higher VFA production and butyrate molar proportion [23]. Fermentation of colostrum proteins and glycerin from triglycerides have also been associated with increments in butyrate production in cows [24]. However, the consistent increase in butyrate across all colostrum sources also points toward a potential modulatory role of bioactive compounds such as IgG and lactoferrin, which have been reported to influence microbial populations and fermentation pathways [5,6]. Supporting this hypothesis, Hyrslova et al. [22] reported that bovine colostrum components can exert prebiotic effects, stimulating the proliferation of beneficial bacteria, leading to increased lactic acid production and reduced acetate proportions. Similarly, previous in vitro work has shown that salivary immunoglobulins, mostly IgA and IgG, can enhance rumen fermentation efficiency and shift fermentation patterns toward propionate and butyrate production [25]. These findings are consistent with the reductions in acetate production per unit of substrate observed in the present study following supplementation of the high dose of OC (−29%), BC (−21%), and RBC (−30%). Collectively, these results suggest that both salivary and colostrum IgG may exert similar modulatory effects towards a butyrate-producing microbiota in the rumen. From a physiological perspective, the increase in butyrate production may be particularly relevant during the weaning transition, given its central role not only on rumen epithelial development but also indirectly on the lower gastrointestinal tract development, including the abomasum and small intestine [26]. Unfortunately, rumen papilla development could not be evaluated in our in vitro study.

The increase in branched-chain VFA and NH_3_-N concentrations further suggests that colostrum represents a significant source of rumen-degradable protein. At low inclusion levels, the proportional increase in the protein supply (1.24-folds higher) and its degradation products, including NH_3_-N (1.09 to 1.12-folds), isobutyrate (1.13 to 1.31-folds) and isovalerate (1.13 to 1.29-folds), suggests efficient microbial utilization. In contrast, at higher doses, the increase in these metabolites (1.37- to 1.49-fold) was proportionally lower than the increase in protein supply (2.20-fold), suggesting that a fraction of colostrum protein may have escaped ruminal degradation under these in vitro conditions. Similar findings have been previously reported for certain milk-derived proteins [27]. However, further in vivo studies would be required to confirm the extent to which these effects may contribute to post-ruminal amino acid supply and animal performance.

In this context, the potential role of colostrum-derived bioactive compounds beyond the rumen should be considered. Compounds such as IgG, lactoferrin, and growth factors may retain biological activity after gastric partial digestion and contribute to intestinal health and microbiota modulation. As a result, there is increasing interest in developing “extended colostrum feeding” strategies for up to 3 weeks, as such approaches have been associated with improved intestinal health, growth performance, and reduced morbidity in young ruminants [28,29,30]. Our findings suggest that this extended colostrum feeding may also provide additional benefits on rumen function, although this hypothesis requires further in vivo validation.

Overall, the present results support the concept that whole colostrum supplementation can stimulate rumen fermentation during the post-weaning period, primarily through increased nutrient supply, but also via modulation of microbial activity. To further elucidate these mechanisms, a colostrum fractionation procedure was developed to disentangle this dual mode of action by assessing the effects after a sequential removal of specific colostrum components.

### 4.2. Effects of Colostrum Defatting

The initial centrifugation step (F1 to F2) effectively removed lipids to undetectable levels while preserving the protein profile and overall fermentative response, as no substantial differences were observed between whole and defatted colostrum. This indicates that lipids were not a major determinant of the baseline stimulatory effect of colostrum on rumen fermentation under the conditions tested. Notably, the efficiency of lipid removal was consistent across colostrum sources despite marked differences in their initial fat content, suggesting that the centrifugation protocol was robust and did not introduce source-dependent bias, being an aspect that justifies its broader applicability.

As observed for whole colostrum, defatted colostrum (F2) increased GP (up to +19%), total VFA concentration (up to +18%), and butyrate proportion (up to +12%) relative to the CTL, confirming that the fermentative response was largely driven by non-lipid components. However, the greater increase in total VFAs observed at higher inclusion levels compared with whole colostrum suggests that lipids may exert a modest inhibitory effect on microbial activity. This interpretation is consistent with the known antimicrobial properties of unsaturated fatty acids present in colostrum, including oleic (C16:1), palmitoleic (C16:1), linoleic (C18:2) and α-linolenic acids [31]. Their removal may therefore alleviate constraints on microbial growth, resulting in enhanced fermentation as previously suggested [32,33]. Despite this, the absence of changes in VFA profile between F1 and F2 indicates that lipids play a limited role in shaping fermentation pathways. This may be related to the relatively low lipolytic and biohydrogenation activity of rumen microbiota, which could restrict the metabolic impact of dietary lipids [34]. Therefore, while lipids appear to influence the magnitude of fermentation, their role in directing fermentation patterns seems minimal under these experimental conditions.

From a nutritional perspective, lipids can represent a substantial fraction of the ruminant colostrum, ranging from 6 to 20% depending on the colostrum source [20], and can represent a key source of energy in newborn ruminants; however, their relevance as an energy source is likely limited when colostrum is used as a feed additive at low inclusion levels during post-weaning. Moreover, the potential inhibitory effects of lipids on rumen microbial activity, together with evidence linking certain lipid fractions to oxidative stress and intestinal inflammatory responses [35], suggest that lipid removal could enhance the functional value of colostrum-derived products as well as their stability. However, these interpretations should be made cautiously, as in vitro systems may overestimate inhibitory effects due to the absence of absorption, passage, and host regulatory mechanisms.

### 4.3. Effect of Immunoglobulins Depletion

A subsequent filtration using a 100 kDa molecular weight cut-off membrane (F2 to F3) led to a marked reduction in total protein content (approximately 86–93%) and the nearly complete removal of high-molecular-weight proteins, mostly IgG (150 kDa), but likely also IgD (180 kDa), IgE (200 kDa) and casein micelles (10^9^ kDa) due to their higher molecular weight [20]. This filtration step was accompanied by substantial decreases in IGF-I (–80%) and lactose (–60%), despite their relatively low molecular weights. This effect may be attributed to their association with larger molecular complexes, such as IGF-binding proteins or glycoprotein structures [36]. Despite the removal of high-molecular-weight components, fraction F3 retained proteins of intermediate and low molecular weight, including lactoferrin (80 kDa), lactoperoxidase (78 kDa), MFGE8 (45 kDa) and EGF (6 kDa).

Interestingly, this 100 kDa filtration led to strong differences in rumen fermentation, indicating that IgG is a key colostrum component when used as feed additive. On one hand, elimination of IgG led to a decrease in the GP (−5.9%, −4.8% and −3.9%) and VFA concentration (−3.1%, −15% and −6.5%) when supplementing high doses of OC, BC and RBC, respectively. This decline in rumen fermentation could be partially explained by the fact that colostrum IgG acts as a fermentable substrate in the rumen, particularly when supplemented at high doses. This would explain the increase in GP per unit of substrate when colostrum IgG was removed because F3 had 4 times lower DM content than F2. Moreover, filtration through 100 kDa not only removed IgG but also led to a significant decrease (−86% to −93%) in the total protein content, as evidenced by an important decline in the concentration of protein-degradation products such as NH_3_-N, isobutyrate and isovalerate for OC (−34% to −35%), BC (−28% to −30%) and RBC (−25% to −30%) when supplemented at high doses, leading to values similar to those observed in the CTL.

Beyond their role as a protein source, colostrum IgG may contribute to the modulation of the rumen microbiota, thereby influencing fermentation patterns, as previously suggested for salivary IgA [37]. In the present study, IgG depletion in F3 was associated with a shift in VFA profiles characterized by an increase in propionate molar proportion (–7.5%, +6.1% and +11% for OC, BC and RBC, respectively) together with a lower rumen pH compared to F2. This pattern is consistent with a greater predominance of amylolytic microbes and may indicate an increased risk of subacute ruminal acidosis under high supplementation conditions. In contrast, the presence of IgG was associated with higher proportions of butyrate and branched-chain VFAs, suggesting a shift towards greater utilization of structural carbohydrates and enhanced proteolytic activity. This IgG microbial modulatory mechanism is consistent with our previous observations, where salivary immunoglobulins enhanced rumen fermentation as supplementation with 0.25 µm filtered saliva increased GP and total VFA concentration compared to autoclaved saliva, whereas these effects were lost following immunoglobulin depletion at 30 kDa [25].

In addition to the positive effects on rumen fermentation and considering that up to 19% of bovine IgG can escape gastric digestion in humans [38], colostrum IgG may also exert downstream effects on intestinal health during the weaning transition in calves. Although systemic absorption of IgG declines sharply after the first 24 h of life, orally administered IgG remains biologically active within the gastrointestinal tract, where it may contribute to the neutralization of enteric pathogens, reduction in epithelial damage, attenuation of mucosal immune activation and inflammation, and promotion of hindgut fermentation [39]. However, the extent to which intact IgG reaches the lower gut in calves and whether the concentration is sufficient to elicit consistent biological effects require further research.

The higher butyrate proportions observed with F3 supplementation (+4.5% to +13%) than the CTL indicate that this fraction still contains bioactive compounds capable of modulating fermentation. Lactoferrin (80 kDa) is a plausible contributor, given its iron-binding capacity and antimicrobial activity, which can selectively constrain the growth of certain Gram-negative and proteolytic bacteria [40]. This interpretation is consistent with recent in vitro evidence showing that moderate lactoferrin supplementation (16.6 and 33.3 mg/L) alters rumen microbial activity, increasing GP and shifting fermentation towards higher efficiency characterized by increased propionate and butyrate [41]. The lactoferrin concentrations achieved in the present study fall within a comparable range. However, attributing these effects specifically to lactoferrin remains speculative, as other co-existing compounds may contribute. Other medium-size proteins such as lactoperoxidase (78 kDa) may also play a role through antimicrobial mechanisms, particularly via the generation of hypothiocyanite [42]. Existing studies suggest broader physiological effects of lactoferrin and lactoperoxidase on the intestine, including increased microbial diversity and reduced inflammation and diarrhea in calves [43,44]; however, their effects on rumen fermentation have not yet been established.

Taken together, our findings support the view that IgG is a key functional component of colostrum that contributes to its overall bioactivity and should be preserved in colostrum-derived products intended for the weaning period. Future studies should focus on optimizing fractionation strategies to selectively enrich these compounds while maintaining their activity.

### 4.4. Effect of Depletion of Medium-Size Molecules

The final ultrafiltration step (10 kDa) from F3 to F4 induced a further ~50% reduction in protein concentration, largely reflecting the removal of intermediate-sized proteins such as lactoferrin (80 kDa; –97%). In contrast, smaller components, including IGF-I, EGF and lactose, were largely retained [39]. Despite these compositional changes, the transition from F3 to F4 had only a limited impact on overall rumen fermentation, suggesting that medium-size proteins such as lactoferrin and lactoperoxidase play a comparatively minor role as direct modulators of rumen microbial activity relative to other colostrum fractions such as IgG.

Interestingly, F4 supplementation still resulted in lower rumen pH and increased butyrate proportions (up to +10%) than the CTL, particularly at higher inclusion levels. This suggests that small, readily fermentable substrates retained in this fraction, such as lactose, small peptides and oligosaccharides, may sustain microbial activity despite the removal of larger proteins. These compounds are known to serve as fermentable substrates and may also exert indirect effects on microbial composition [22]. In this line, a recent study showed that supplementation of calves with specific colostrum oligosaccharides (i.e., 3′-sialyllactose and 6′-sialyllactose) increased DMI before weaning, modulated the intestinal microbiota, and improved the intestinal barrier development [45]. The presence of small-size bioactive molecules such as lysozyme (14 kDa) and growth factors (e.g., EGF) could also potentially modulate the rumen microbiota [40]; however, their effects on the rumen fermentation remain unknown. Moreover, the concentrations of lactoperoxidase, lysozyme, and oligosaccharides were not determined in the present study, limiting our ability to infer their specific contribution to the observed fermentation responses. Future research should incorporate quantitative characterization of these components and employ longer-term experimental approaches (e.g., Rusitec or dual-flow fermenters) or in vivo trials to better elucidate their biological relevance and mechanisms of action.

## 5. Conclusions

Overall, the present in vitro study suggests that colostrum-derived products may contribute to rumen fermentation not only by providing fermentable nutrients but also through bioactive effects capable of modulating microbial activity. The consistency of the responses among colostrum sources indicates a potential practical interchangeability, whereas the fractionation approach highlights the relevance of preserving specific bioactive components during product processing. In particular, IgG appeared to play a relevant functional role in maintaining a balanced fermentation pattern in vitro, while lipid removal showed potential advantages for product stability without compromising fermentative responses. These findings provide new insight into the potential application of IgG-enriched colostrum products as nutritional strategies to enhance rumen fermentation in young ruminants. However, the relevance of these findings under practical feeding conditions during the weaning transition should be confirmed through dedicated in vivo studies before definitive conclusions or recommendations can be made.

## Figures and Tables

**Table 1:** Composition of different colostrum sources and fractions (in FM).

		Fractions			
	Source ^1^	F1	F2	F3	F4
DM (in % FM)	OC	25.0 ± 1.16	18.5 ± 0.08	5.29 ± 0.74	4.02 ± 0.71
	BC	19.4 ± 0.08	15.0 ± 0.21	6.20 ± 0.83	4.95 ± 0.97
	RBC	19.4 ± 0.44	15.8 ± 0.25	4.05 ± 1.00	2.95 ± 0.06
Brix (%)	OC	21.1 ± 0.04	21.0 ± 0.08	6.90 ± 0.03	4.90 ± 0.03
	BC	18.0 ± 0.14	17.4 ± 0.16	7.40 ± 0.04	5.40 ± 0.04
	RBC	19.9 ± 0.08	18.9 ± 0.10	5.40 ± 0.03	4.60 ± 0.02
Fat (g/L)	OC	86.6 ± 1.55	ND	ND	ND
	BC	50.3 ± 4.50	ND	ND	ND
	RBC	53.6 ± 2.11	ND	ND	ND
Proteins (g/L)	OC	99.7 ± 2.83	97.4 ± 3.26	13.6 ± 0.18	5.44 ± 0.29
	BC	97.3 ± 2.71	96.6 ± 3.04	9.35 ± 0.24	4.03 ± 0.31
	RBC	100 ± 7.46	101 ± 3.90	6.90 ± 0.19	4.93 ± 0.43
Lactose (g/L)	OC	61.1 ± 2.80	76.7 ± 3.01	31.2 ± 1.02	30.0 ± 1.16
	BC	85.0 ± 3.99	86.7 ± 3.20	41.2 ± 2.25	30.6 ± 2.06
	RBC	77.2 ± 4.25	86.9 ± 3.80	30.3 ± 1.50	24.4 ± 1.94
IgG Brix (g/L, BRIX)	OC	59.6 ± 3.58	57.1 ± 3.49	ND	ND
	BC	35.0 ± 2.12	36.7 ± 2.21	ND	ND
	RBC	35.6 ± 2.16	45.2 ± 2.70	ND	ND
IgG ELISA (g/L)	OC	38.3 ± 2.91	34.3 ± 3.01	1.13 ± 0.05	1.06 ± 0.04
	BC	14.4 ± 2.30	13.1 ± 2.03	0.41 ± 0.03	0.38 ± 0.02
	RBC	13.7 ± 0.60	12.6 ± 2.06	0.38 ± 0.02	0.34 ± 0.01
Lactoferrin (g/L)	OC	NM	NM	NM	NM
	BC	4.48 ± 0.73	4.60 ± 1.34	3.06 ± 0.05	0.09 ± 0.04
	RBC	4.43 ± 1.01	3.73 ± 0.85	2.75 ± 0.01	0.10 ± 0.05
MFGE8 (µg/L)	OC	105 ± 2.57	103 ± 1.65	139 ± 1.73	147 ± 4.52
	BC	101 ± 0.65	102 ± 0.54	143 ± 3.54	152 ± 11.0
	RBC	112 ± 9.17	109 ± 2.90	149 ± 5.06	176 ± 5.81
IGF-I (µg/L)	OC	153 ± 7.55	136 ± 6.03	24.3 ± 1.68	24.1 ± 1.62
	BC	105 ± 21.0	105 ± 2.00	21.3 ± 3.00	20.9 ± 0.62
	RBC	108 ± 18.9	109 ± 15.7	19.4 ± 1.47	17.7 ± 0.69
EGF (ng/L)	OC	461 ± 33.8	593 ± 27.1	693 ± 27.7	932 ± 51.6
	BC	467 ± 73.8	428 ± 9.40	827 ± 24.3	943 ± 29.3
	RBC	435 ± 10.2	429 ± 24.3	675 ± 8.70	997 ± 8.70

^1^ Abbreviations: OC: ovine colostrum; BC, bovine colostrum; RBC, reconstituted bovine colostrum, MFGE8, milk fat globule EGF 8; EGF, epidermal growth factor; ND, not detected; NM, not measured. Mean and standard deviation based on three samples.

**Table 2:** Effects of incubation with low (4 mL/L) or high (20 mL/L) doses of different sources of whole colostrum on in vitro fermentation using rumen inocula from weaned lambs.

	Low Doses						High Doses					
	CTL	OC	BC	RBC	SEM ^1^	*p*-Value	CTL	OC	BC	RBC	SEM ^1^	*p*-Value
Rumen fermentation												
GP (mL)	140 ^a^	153 ^c^	148 ^bc^	145 ^b^	1.506	0.001	140 ^a^	172 ^c^	164 ^b^	161 ^b^	1.672	<0.001
CH_4_ (mL)	3.68 ^a^	4.46 ^c^	4.01 ^b^	4.09 ^ab^	0.109	0.006	3.68 ^a^	5.19 ^b^	5.29 ^b^	5.03 ^b^	0.097	<0.001
pH	6.28	6.25	6.25	6.24	0.013	0.221	6.28 ^b^	6.20 ^a^	6.21 ^a^	6.17 ^a^	0.012	0.001
NH_3_-N (mg/dL)	60.8 ^a^	68.4 ^c^	66.3 ^b^	66.3 ^b^	0.749	<0.001	60.8 ^a^	90.6 ^c^	84.5 ^b^	83.4 ^b^	1.535	<0.001
VFAs (mmol/L)	122	119	130	116	9.099	0.757	122	135	135	126	7.929	0.601
Acetate (%)	41.1	38.8	39.3	38.3	1.147	0.379	41.1	38.9	39.8	39.2	1.244	0.606
Propionate (%)	40.5	41.0	41.0	39.4	0.867	0.549	40.5	38.6	37.2	37.0	1.319	0.271
Butyrate (%)	9.95	10.6	10.5	11.6	0.485	0.163	9.95 ^a^	10.6 ^ab^	11.3 ^b^	11.0 ^b^	0.344	0.036
Isobutyrate (%)	1.66 ^a^	1.92 ^ab^	1.92 ^ab^	2.27 ^b^	0.124	0.044	1.66 ^a^	2.33 ^b^	2.45 ^b^	2.54 ^b^	0.099	0.001
Valerate (%)	4.05	4.57	4.30	4.78	0.254	0.263	4.05 ^a^	5.80 ^b^	5.44 ^b^	5.68 ^b^	0.238	0.002
Isovalerate (%)	2.68	3.10	3.03	3.62	0.222	0.086	2.68 ^a^	3.75 ^b^	3.83 ^b^	4.02 ^b^	0.173	0.002
Fermentation products (per g DM)												
GP (ml)	280	278	276	270	2.830	0.165	280 ^b^	230 ^a^	237 ^a^	233 ^a^	2.920	<0.001
CH_4_ (ml)	7.37	8.10	7.44	7.58	0.203	0.111	7.37	6.92	7.63	7.25	0.168	0.085
NH_3_-N (mg)	3.95 ^a^	4.28 ^b^	4.18 ^b^	4.18 ^b^	0.070	0.049	3.95 ^a^	4.62 ^b^	4.55 ^b^	4.47 ^b^	0.125	0.016
VFAs (mmol)	8.20	7.22	8.35	7.11	0.847	0.643	8.20	6.32	6.84	6.24	0.584	0.134
Acetate (mmol)	3.65	2.98	3.47	2.92	0.388	0.498	3.65 ^b^	2.58 ^a^	2.88 ^ab^	2.56 ^a^	0.252	0.045
Propionate (mmol)	2.98	2.66	3.11	2.48	0.405	0.688	2.98	2.15	2.22	2.00	0.316	0.197
Butyrate (mmol)	0.83	0.80	0.91	0.85	0.054	0.520	0.83	0.71	0.81	0.77	0.046	0.311
Isobutyrate (mmol)	0.15	0.16	0.18	0.19	0.011	0.114	0.15	0.17	0.20	0.19	0.012	0.053
Valerate (mmol)	0.35	0.37	0.38	0.37	0.011	0.412	0.35 ^a^	0.43 ^b^	0.42 ^b^	0.41 ^b^	0.012	0.008
Isovalerate (mmol)	0.24	0.26	0.29	0.30	0.014	0.075	0.24 ^a^	0.28 ^ab^	0.31 ^b^	0.30 ^b^	0.017	0.069

^1^ Abbreviations: CTL, control; OC: ovine colostrum; BC, bovine colostrum; RBC, reconstituted bovine colostrum; SEM, standard error of the mean (*n* = 4). ^a–c^ Means with different letters differ across the same row (*p* < 0.05).

**Table 3:** Effects of incubation with low (4 mL/L) or high (20 mL/L) doses of different colostrum sources and fractions on in vitro fermentation using rumen inocula from weaned lambs.

		Low Dose				High Dose						*p*-Value	
	CTL	F1	F2	F3	F4	F1	F2	F3	F4	SEM ^1^	Frac	Dose	F × D
Total GP (mL)													
OC	140	153 *	152 *	144	142	172 *	167 *	157 *	157 *	1.663	<0.001	<0.001	0.239
BC	140	148 *^bc^	147 ^†ab^	144 ^a^	147 ^ab^	164 *^e^	163 *^e^	152 *^cd^	154 *^d^	1.126	<0.001	<0.001	0.001
RBC	140	145 ^ab^	143 ^a^	142 ^a^	143 ^a^	161 *^c^	161 *^c^	149 *^b^	148 *^b^	1.496	<0.001	<0.001	0.001
CH_4_ (mL)													
OC	3.68	4.47 *	4.18 *	4.07 *	4.00 ^†^	5.19 *	5.01 *	4.67 *	4.58 *	0.125	0.001	<0.001	0.744
BC	3.68	4.01 ^†a^	4.44 ^†a^	4.08 *^a^	4.33 *^a^	5.29 *^b^	5.14 *^b^	4.31 *^a^	4.43 *^a^	0.157	0.005	<0.001	0.005
RBC	3.68	4.09 ^†bc^	3.95 ^†abc^	3.79 ^ab^	3.79 ^a^	5.03 *^e^	5.12 *^e^	4.53 *^d^	4.23 *^c^	0.102	<0.001	<0.001	0.012
pH													
OC	6.28	6.25 ^d^	6.24 ^cd^	6.24 ^cd^	6.23 ^†c^	6.20 *^b^	6.24 ^cd^	6.15 *^a^	6.16 *^a^	0.009	<0.001	<0.001	0.001
BC	6.28	6.25 ^†e^	6.24 *^de^	6.23 ^†cd^	6.22 *^bcd^	6.21 *^b^	6.22 *^bc^	6.17 *^a^	6.19 *^a^	0.006	<0.001	<0.001	0.026
RBC	6.28	6.24 ^†c^	6.23 *^c^	6.22 ^†c^	6.24 *^c^	6.17 *^a^	6.19 *^b^	6.18 *^ab^	6.19 *^b^	0.046	0.119	<0.001	0.049
NH_3_-N (mg/dL)													
OC	60.8	68.4 *^b^	68.6 *^b^	61.6 ^a^	59.8 ^a^	90.6 *^c^	90.5 *^c^	59.8 ^a^	59.4 ^a^	1.362	<0.001	<0.001	<0.001
BC	60.8	66.3 *^b^	67.2 *^b^	59.4 ^a^	59.8 ^a^	84.5 *^c^	85.2 *^c^	60.5 ^a^	60.4 ^a^	1.026	<0.001	<0.001	<0.001
RBC	60.8	66.3 *^b^	64.5 *^b^	61.6 ^a^	60.8 ^a^	83.4 *^c^	85.3 *^c^	60.6 ^a^	60.2 ^a^	0.857	<0.001	<0.001	<0.001

^1^ Abbreviations: CTL, control; OC: ovine colostrum; BC, bovine colostrum; RBC, reconstituted bovine colostrum; F1, whole colostrum; F2, non-fat colostrum; F3, non-fat, non-IgG colostrum; F4, non-fat, non-protein colostrum; SEM, standard error of the mean (*n* = 4). ^a–e^ Means with different letters differ across the same row (*p* < 0.05). *^†^ Pairwise comparisons with the CTL (* *p* < 0.05; ^†^ *p* < 0.1).

**Table 4:** Effects of incubation with low (4 mL/L) or high (20 mL/L) doses of different colostrum sources and fractions on in vitro VFA concentration using rumen inocula from weaned lambs.

		Low Dose				High Dose					*p*-Value		
	CTL	F1	F2	F3	F4	F1	F2	F3	F4	SEM ^1^	Frac	Dose	F × D
VFAs (mmol/L)													
OC	122	119	128	116	127	135	133	129	126	6.724	0.733	0.098	0.610
BC	122	130	128	127	115	135	144 ^†^	122	130	6.131	0.117	0.083	0.300
RBC	122	116	118	114	119	126	140 ^†^	131	133	8.660	0.791	0.018	0.916
Acetate (%)													
OC	41.1	38.8 ^†^	38.4 *	37.6	40.0	38.9	37.4 *	38.7 ^†^	39.0	1.040	0.422	0.755	0.723
BC	41.1	39.3	40.6	44.2	41.0	39.8	41.7	42.5	42.6	1.475	0.111	0.713	0.706
RBC	41.1	38.3	37.6	38.9	36.9	39.2	43.8	43.1	42.1	1.815	0.567	0.004	0.507
Propionate (%)													
OC	40.5	41.0	40.3	41.5	41.1	38.6 *	38.5 *	41.4	41.4	0.545	0.002	0.016	0.055
BC	40.5	41.0 ^c^	38.8 ^bc^	37.1 ^ab^	38.5 ^ab^	37.2 ^ab^	35.9 ^†a^	38.1 ^ab^	38.1 ^ab^	0.869	0.217	0.025	0.039
RBC	40.5	39.4	39.7	39.6	41.5	37.0	34.2	37.9	38.4	1.012	0.052	<0.001	0.300
Butyrate (%)													
OC	9.95	10.6 ^†^	11.0 ^†^	11.2 *	10.3	10.6	11.1 ^†^	11.2 *	10.9 ^†^	0.328	0.230	0.370	0.745
BC	9.95	10.5	10.9	10.2	11.1 ^†^	11.3 *	10.7 ^†^	10.7 ^†^	10.7 ^†^	0.411	0.659	0.561	0.418
RBC	9.95	11.6 ^†^	11.6 ^†^	11.6 ^†^	11.7 ^†^	11.6 *	10.5 *	10.4 *	10.7 ^†^	0.470	0.554	0.022	0.617
Isobutyrate (%)													
OC	1.66	1.92 *^bc^	2.11 *^cd^	2.02 *^c^	1.71 ^ab^	2.33 *^d^	2.60 *^e^	1.72 ^ab^	1.63 ^a^	0.089	<0.001	0.049	<0.001
BC	1.66	1.92 ^ab^	2.07 ^b^	1.74 ^a^	1.93 ^ab^	2.45 *^c^	2.42 *^c^	1.70 ^a^	1.74 ^a^	0.089	<0.001	0.018	0.002
RBC	1.66	2.27 *^cde^	2.32 *^de^	2.01 *^bc^	2.05 *^cd^	2.54 *^e^	2.31 *^de^	1.71 ^a^	1.77 ^ab^	0.095	<0.001	0.247	0.023
Valerate (%)													
OC	4.05	4.57 *^ab^	4.76 ^b^	4.50 ^ab^	4.17 ^a^	5.80 *^c^	6.20 *^c^	4.36 ^ab^	4.38 ^ab^	0.186	<0.001	<0.001	0.001
BC	4.05	4.30 ^a^	4.44 ^a^	4.05 ^a^	4.36 ^a^	5.44 *^b^	5.41 *^b^	4.23 ^a^	4.05 ^a^	0.179	<0.001	0.001	0.002
RBC	4.05	4.78 ^abc^	5.04 ^bcd^	4.62 ^ab^	4.58 ^ab^	5.68 *^d^	5.47 *^cd^	4.14 ^a^	4.15 ^a^	0.242	0.001	0.557	0.024
Isovalerate (%)													
OC	2.68	3.10 *^bc^	3.38 *^cd^	3.25 *^c^	2.72 ^ab^	3.75 *^de^	4.18 *^e^	2.73 ^ab^	2.62 ^a^	0.157	<0.001	0.075	0.001
BC	2.68	3.03 ^ab^	3.20 ^b^	2.75 ^a^	3.08 ^ab^	3.83 *^c^	3.80 *^c^	2.72 ^a^	2.76 ^a^	0.145	<0.001	0.018	0.003
RBC	2.68	3.62 ^†cd^	3.66 ^†cd^	3.24 ^†abc^	3.25 *^bc^	4.02 *^d^	3.66 *^cd^	2.74 ^a^	2.83 ^†ab^	0.169	<0.001	0.296	0.053

^1^ Abbreviations: CTL, control; OC: ovine colostrum; BC, bovine colostrum; RBC, reconstituted bovine colostrum; F1, whole colostrum; F2, non-fat colostrum; F3, non-fat, non-IgG colostrum; F4, non-fat, non-protein colostrum; SEM, standard error of the mean (*n* = 4). ^a–e^ Means with different letters differ across the same row (*p* < 0.05). *^†^ Pairwise comparisons with the CTL (* *p* < 0.05; ^†^ *p* < 0.1).

**Table 5:** Effects of incubation with low (4 mL/L) or high (20 mL/L) doses of different colostrum sources and fractions on in vitro rumen fermentation products (per g DM) in weaned lambs.

		Low Dose				High Dose					*p*-Value		
	CTL	F1	F2	F3	F4	F1	F2	F3	F4	SEM ^1^	Frac	Dose	F × D
Total GP (mL)													
OC	280	278 ^c^	284 ^cd^	282 ^cd^	280 ^c^	230 *^a^	244 *^b^	283 ^cd^	290 ^d^	3.022	<0.001	<0.001	<0.001
BC	280	276 ^cd^	278 ^d^	281 ^de^	287 ^e^	237 *^a^	251 *^b^	270 ^c^	281 ^d^	2.218	<0.001	<0.001	<0.001
RBC	280	270 ^c^	268 ^†c^	280 ^d^	282 ^d^	233 *^a^	244 *^b^	276 ^cd^	280 ^d^	2.641	<0.001	<0.001	<0.001
CH_4_ (mL)													
OC	7.37	8.10 *^cd^	7.79 ^bcd^	7.96 *^bcd^	7.87 ^bcd^	6.92 ^a^	7.31 ^ab^	8.44 *^cd^	8.49 ^†d^	0.230	0.006	0.403	0.002
BC	7.37	7.44	8.39	7.96 *	8.49 *	7.63	7.91	7.68	8.05 *	0.291	0.078	0.230	0.655
RBC	7.37	7.58 ^ab^	7.43 ^a^	7.47 ^ab^	7.49 ^ab^	7.25 ^a^	7.78 ^ab^	8.39 ^†c^	7.98 *^bc^	0.187	0.077	0.013	0.024
NH_3_-N (mg)													
OC	3.95	4.28 *^cd^	4.41 *^d^	3.95 ^bc^	3.79 ^ab^	4.62 ^†d^	5.05 *^e^	3.48 *^a^	3.53 *^a^	0.124	<0.001	0.471	0.001
BC	3.95	4.18 *^c^	4.33 *^cd^	3.72 ^ab^	3.78 ^b^	4.55 *^d^	4.92 *^e^	3.49 *^a^	3.56 *^ab^	0.091	<0.001	0.060	<0.001
RBC	3.95	4.18 ^†c^	4.06 ^bc^	3.96 ^bc^	3.90 ^ab^	4.47 *^d^	4.86 *^e^	3.63 *^a^	3.68 ^a^	0.093	<0.001	0.051	<0.001
Total VFAs (mmol)													
OC	8.20	7.22 *	8.17	7.50	8.57	6.32 *	6.77	8.02	7.94	0.612	0.138	0.178	0.464
BC	8.20	8.35	8.32	8.54	7.33	6.84	8.03	7.35	8.19	0.545	0.742	0.182	0.160
RBC	8.20	7.11	7.39	7.31	7.86	6.24	7.64	8.46	8.84	0.874	0.296	0.552	0.653
Acetate (mmol)													
OC	3.65	2.98 *	3.33	2.95	3.63	2.58 *	2.62 ^†^	3.29	3.29	0.311	0.194	0.222	0.410
BC	3.65	3.47	3.66	4.21	3.30	2.88	3.56	3.41	3.84	0.349	0.344	0.345	0.249
RBC	3.65	2.92	2.93	3.11	3.09	2.56 *	3.61	3.96	4.05	0.502	0.353	0.147	0.548
Propionate (mmol)													
OC	2.98	2.66 *	2.96	2.80	3.20	2.15 *	2.29	3.04	2.90	0.261	0.067	0.136	0.343
BC	2.98	3.11	2.87	2.67	2.42	2.22	2.53	2.42	2.72	0.204	0.841	0.052	0.063
RBC	2.98	2.48	2.66	2.54	2.98	2.00	2.18	2.81	3.01	0.321	0.132	0.476	0.560
Butyrate (mmol)													
OC	0.83	0.80 *	0.93	0.90	0.93	0.71 *	0.80	0.92	0.91	0.054	0.019	0.167	0.512
BC	0.83	0.91	0.90	0.88	0.84	0.81	0.87	0.83	0.89	0.041	0.871	0.222	0.336
RBC	0.83	0.85	0.89	0.86	0.94	0.77	0.83	0.90	0.95	0.057	0.161	0.608	0.679
Isobutyrate (mmol)													
OC	0.15	0.16	0.20 ^†^	0.18	0.16	0.17	0.22 ^†^	0.15	0.14	0.010	<0.001	0.552	0.118
BC	0.14	0.18	0.20	0.16	0.16	0.20	0.23 ^†^	0.14	0.15	0.011	<0.001	0.604	0.079
RBC	0.14	0.19	0.20	0.17	0.19	0.19	0.21 *	0.16	0.17	0.012	0.012	0.586	0.734
Valerate (mmol)													
OC	0.35	0.37 ^†^	0.43 *	0.38	0.38	0.43 *	0.49 *	0.37	0.37	0.018	<0.001	0.053	0.087
BC	0.35	0.38 ^bc^	0.38 ^bc^	0.36 ^ab^	0.34 ^ab^	0.42 *^c^	0.48 ^†d^	0.33 ^a^	0.34 ^ab^	0.017	<0.001	0.027	0.008
RBC	0.35	0.37	0.40	0.37	0.37	0.4 ^†^	0.48 *	0.37	0.35	0.018	0.005	0.012	0.165
Isovalerate (mmol)													
OC	0.24	0.26	0.32 ^†^	0.29	0.26	0.28	0.35 ^†^	0.24	0.23	0.015	<0.001	0.468	0.067
BC	0.24	0.29 ^†^	0.31	0.26	0.26	0.31	0.36 ^†^	0.23	0.25	0.017	<0.001	0.568	0.085
RBC	0.24	0.30	0.32	0.27	0.30	0.30	0.33 *	0.25	0.27	0.015	0.005	0.678	0.587

^1^ Abbreviations: CTL, control; OC: ovine colostrum; BC, bovine colostrum; RBC, reconstituted bovine colostrum; F1, whole colostrum; F2, non-fat colostrum; F3, non-fat, non-IgG colostrum; F4, non-fat, non-protein colostrum; SEM, standard error of the mean (*n* = 4). ^a–e^ Means with different letters differ across the same row (*p* < 0.05). *^†^ Pairwise comparisons with the CTL (* *p* < 0.05; ^†^ *p* < 0.1).

## Data Availability

Data are available on request by contacting the corresponding author.
